# Angiography-derived physiological patterns of coronary artery disease: implications with post-stenting physiology and long-term clinical outcomes

**DOI:** 10.1007/s00392-024-02500-8

**Published:** 2024-08-05

**Authors:** Simone Fezzi, Paolo Alberto Del Sole, Francesco Burzotta, Antonio Maria Leone, Daixin Ding, Dimitrios Terentes-Printzios, Carlo Trani, Luca Bonizzi, Sara Sgreva, Stefano Andreaggi, Jiayue Huang, Gabriele Pesarini, Domenico Tavella, Guy Prado, Andrea Vicerè, Dimitrios Oikonomou, Konstantia Paraskevi Gkini, Domenico Galante, Konstantinos Tsioufis, Charalambos Vlachopoulos, William Wijns, Flavio Ribichini, Shengxian Tu, Roberto Scarsini

**Affiliations:** 1 Division of Cardiology, Department of Medicine, University of Verona, Piazzale A. Stefani 1, Verona, Italy; 2 The Smart Sensors Laboratory and Curam, The Lambe Institute for Translational Medicine, Univesity of Galway, Galway, Ireland; 3 Fondazione Policlinico Universitario A. Gemelli IRCCS, Rome, Italy; 4 Shanghai Jiao Tong University School of Medicine Affiliated Ren Ji Hospital, Shanghai, China; 5 First Department of Cardiology, Medical School, Hippokration Hospital, National and Kapodistrian University of Athens, Athens, Greece; 6 Department of Clinical and Molecular Medicine, Sapienza University, Rome, Italy; 7 Biomedical Instrument Institute, School of Biomedical Engineering, Shanghai Jiao Tong University, Shanghai, China; 8 Università Cattolica del Sacro Cuore, Rome, Italy

**Keywords:** Angiography-derived physiology, Quantitative flow ratio, Physiological pattern of coronary disease, Intracoronary imaging, Percutaneous coronary intervention

## Abstract

**Background:**

Physiological patterns of coronary artery disease (CAD) have emerged as potential determinants of functional results of percutaneous coronary interventions (PCI) and of vessel-oriented clinical outcomes (VOCE).

**Objectives:**

In this study, we evaluated the impact of angiography-derived physiological patterns of CAD on post-PCI functional results and long-term clinical outcomes.

**Methods:**

Pre-PCI angiography-derived fractional flow reserve (FFR) virtual pullbacks were quantitatively interpreted and used to determine the physiological patterns of CAD. Suboptimal post-PCI physiology was defined as an angiography-derived FFR value ≤ 0.91. The primary endpoint was the occurrence of VOCE at the longest available follow-up.

**Results:**

Six hundred fifteen lesions from 516 patients were stratified into predominantly focal (*n* = 322, 52.3%) and predominantly diffuse (*n* = 293, 47.7%). Diffuse pattern of CAD was associated with lower post-PCI angiography-derived FFR values (0.91 ± 0.05 vs. 0.94 ± 0.05; *p* = 0.001) and larger rate of suboptimal post-PCI physiology (43.0 vs. 22.7%; *p* = 0.001), as compared to focal CAD. At the median follow-up time of 37 months (33–58), post-PCI suboptimal physiology was related to a higher risk of VOCE (16.2% vs. 7.6%; HR: 2.311; 95% CI 1.410–3.794; *p* = 0.0009), while no significant difference was noted according to baseline physiological pattern. In diffuse disease, the use of intracoronary imaging was associated with a lower incidence of long-term VOCE (5.1% vs 14.8%; HR: 0.313, 95% CI 0.167–0.614, *p* = 0.030).

**Conclusions:**

Suboptimal post-PCI physiology is observed more often in diffusely diseased arteries and it is associated with higher risk of VOCE at follow-up. The use of intravascular imaging might improve clinical outcomes in the setting of diffuse CAD.

**Supplementary Information:**

The online version contains supplementary material available at 10.1007/s00392-024-02500-8.

## Introduction

Post-PCI suboptimal physiology is associated with reduced quality-of-life improvement and worse clinical outcomes. Despite being very common, it is seldom investigated and poorly recognized [3–5]. Physiological patterns of CAD (i.e., focal, diffuse, mixed, and serial) have recently been shown to potentially impact on PCI outcomes. Pressure wire (PW)-pullback has the potential to qualitatively interpret the physiological pattern of CAD. However, this method lacks of standardization and reproducibility, especially when dealing with ambiguous lesions, on top of requiring a dedicated pressure-wire. To overcome such limitations, the pullback pressure gradient index (PPGi) and the instantaneous fractional flow reserve (FFR) gradient per unit time (dFFR[t]/dt) have been introduced, both calculated based on PW-pullback performed during continuous hyperaemia. PPGi is a continuous metric that serves as a quantitative measure of the physiological distribution of coronary plaques along the vessel and is capable of distinguishing between focal and diffuse disease, while dFFR[t]/dt reflects the local physiological disease severity [6–10].

Despite the extensive evidence in support of physiological assessment as a gate-keeper for coronary revascularization, and the growing one in support of physiology guidance for PCI optimization, its use remains hampered by economic and logistic reasons (i.e., need for a PW and for hyperaemic agents, increased costs and procedural time). To overcome such limitations, angiography-derived physiological indices, such as the quantitative flow ratio (QFR), have been recently developed and validated [11–14]. However, the prognostic value of the functional angiography-based longitudinal analysis, including the QFR-based virtual pullback index (QVPi) and the dQFR/ds, is not fully understood [15, 16].

Therefore, this study aimed at defining the interaction of pre-PCI physiological pattern of CAD, defined according to the QVPi, and local disease severity, defined according to the dQFR/ds, with post-PCI physiological result. Moreover, we sought to assess the association between functional angiography-derived metrics and the incidence of VOCE at long-term follow-up.

## Methods

### Study population

This is a patient-level pooled analysis of four prospective cohorts of patients with de-novo CAD that underwent clinically indicated PCI, enrolled in three European centers (Verona University Hospital, Verona, Italy [185 CESC]; Policlinico Gemelli, Rome, Italy [PROPHET-FFR NCT05056662 and FORZA NCT01824030]; Hippokration Hospital, Athens) between December 2012 and September 2020. Coronary angiograms were anonymized, transferred, and analyzed in two independent laboratories (University of Verona, Italy; The Lambe Institute of Translational Research, University of Galway, Ireland) in a blinded fashion, by experienced and certified analyzers.

Patients younger than 18 years old, with known contraindication to dual anti-platelet therapy, a concomitant indication to open-heart surgery, presenting with resuscitated cardiac arrest and women with childbearing potential were excluded from the study. Furthermore, patients with unavailable pre-PCI and post-PCI coronary angiograms and those with angiographic features limiting either pre-PCI or post-PCI angiography-derived FFR computation (ostial left main or ostial right coronary artery, ongoing ventricular arrhythmias or significant and persistent tachycardia, poor angiography image quality, and severe tortuosity) were excluded from the analysis.

The study was conducted in accordance with the ethical principles of the Declaration of Helsinki, and it was approved by the institutional ethical board of each referring hospital. All the patients had provided their written consent for the anonymous data collection.

### Percutaneous coronary intervention

Invasive coronary angiography and PCI were performed according to the best local practice and to standardized technical recommendations. Particularly, intracoronary nitrates (100–300 mcg) were administered in all cases. Standard radial or femoral access with 5 or 6F catheters were used. Intravascular imaging pre- and post-PCI, such as intravascular ultrasound (IVUS) and optical coherence tomography (OCT), was left to operator discretion. Patients enrolled in the FORZA trial were randomly assigned to either FFR- or OCT-guidance during PCI.

All the patients received dual anti-platelet therapy based on aspirin combined with either clopidogrel, ticagrelor, or prasugrel for at least 12 months in case of acute coronary syndrome or 6 months in case of chronic coronary syndrome, according to the international recommendations. In patients requiring anticoagulation, a direct oral anticoagulant combined with aspirin and clopidogrel for 7–28 days and clopidogrel for 12 months was recommended.

### Quantitative flow ratio and Murray law-based quantitative flow ratio analysis

Quantitative coronary angiography was performed and minimum lumen diameter, reference vessel size, percent diameter stenosis, and lesion length were measured. Estimation of angiography-derived FFR was based on single-view Murray law-based quantitative flow ratio analysis (μFR) and on 3D quantitative flow ratio (QFR). Computational analysis of μFR was performed with the AngioPlus Core, version V3 software (Pulse Medical, Shanghai, China), while of 3D-QFR with the Medis Medical Imaging Systems software (Leiden, The Netherlands), respectively. Analyzers were blinded to any clinical data. Methodologies for 2D-μFR and 3D-QFR were adopted as previously reported [12, 17] and are reported in the Supplementary material. Post-PCI assessment was performed on the angiographic projection acquired at the end of the procedure. Conventional validated cut-off values for ischemia pre-PCI (QFR ≤ 0.80 and μFR ≤ 0.80) and suboptimal physiology post-PCI (QFR ≤ 0.91 and μFR ≤ 0.91) were adopted [18, 19].

### Physiological pattern characterization

Physiological patterns of CAD were classified, based on QFR or μFR virtual pullback trace. More specifically, physiological distribution was assessed through the QFR-virtual pullback pressure gradient index (QVPi) which discriminates predominantly focal from predominantly diffuse disease, providing a continuous metric based on the magnitude of maximum pressure drop over 20 mm and on the extent of functional disease over the entire interrogated vessel.

QVPi was calculated as follows:

$$\frac{{\left[ {\frac{{Max{\mkern 1mu} QVP_{{20mm}} }}{{\Delta QFR_{{vessel}} }} + \left( {1 - \frac{{Lenght{\mkern 1mu} with{\mkern 1mu} functional{\mkern 1mu} disease_{{\left( {mm} \right)}} }}{{Total{\mkern 1mu} vessel{\mkern 1mu} lenght_{{\left( {mm} \right)}} }}} \right)} \right]}}{2}$$

As previously reported, high QVPi values (close to 1) suggest predominantly focal disease, whereas low values (close to 0) predominantly diffuse disease. The median QVPi value (0.68) was used to dichotomize CAD into focal (QVPi ≥ 0.68) and diffuse (QVPi < 0.68) disease [17].

Next, the local physiological severity was calculated by the instantaneous QFR gradient per unit length (dQFR/ds), using a cut-off value of 0.025/mm to identify the presence (≥ 0.025/mm) or the absence (< 0.025/mm) of major gradients, as previously validated [17].

### Follow-up and outcomes

The occurrence of any procedural-related clinical complication was prospectively evaluated. After discharge, follow-up was prospectively conducted during outpatients’ clinic visits or with telephonic interviews and clinical controls and confirmed with medical records’ consultation. Clinical endpoints were assessed at the longest follow-up available.

The target vessel was defined as the entire major coronary vessel proximal and distal to the target lesion, including upstream and downstream branches and the target lesion itself. All clinical outcomes were defined according to the Academic Research Consortium [20]. Vessel-oriented composite endpoint (VOCE) was defined as a composite of cardiac death, target vessel-related myocardial infarction, clinically driven target vessel revascularization (TVR). All deaths were considered cardiac unless an undisputed non-cardiac cause could be demonstrated.

### Statistical analysis

Continuous variables are presented as mean and standard deviation if normally distributed and compared with unpaired t test. Categorical data are reported as a percentage and compared with the *χ*^2^ test or Fisher exact test as appropriate. Correlation coefficients were calculated to assess the relationships of QVPi and dQFR/ds with post-PCI angiography-derived FFR and percentage of angiography-derived FFR increase (Pearson or Spearman according to normality). Mean values and percentage increase of post-PCI angiography-derived FFR were compared between predominant focal and diffuse disease groups, as well as major gradient and no major gradient groups using Student’s t tests. Logistic regression was performed to assess predictors of suboptimal functional PCI results. Survival analysis was performed using Kaplan–Meier methods and groups were compared with log-rank test. Cox regression analysis was performed to assess significant predictors of VOCE. Hazard ratio with 95% confidence interval were provided. Shared frailty Cox regression multivariable analysis, with patient identification introduced in a multilevel model, was performed to take into account the nonindependence of lesions. All analyses were performed with IBM® SPSS® Statistics (Version 26, SPSS Inc., Chicago, IL). Graphics were realized with GraphPad Prism 7.0.

## Results

### Baseline characteristics

Overall, 615 coronary vessels (*n* = 516 patients) underwent PCI with implantation of at least one drug eluting stent (DES; *n* = 427) or a bio-resorbable scaffold (BRS; *n* = 188) and were included in this analysis (Supplementary Fig. 1). The mean age was 65.2 ± 12.6 years, and the majority of the patients were males (416 patients; 67.7%) and presented with chronic coronary syndromes (441 patients; 71.7%). Multivessel CAD was present in 282 patients (45.9%).

Complete baseline characteristics of all patients are displayed in Table 1**.**

**Table 1:** Baseline and procedural characteristics

Clinical features (*n* = 615)	Overall (*n* = 615)	Focal pattern (*n* = 322, 52.3%)	Diffuse pattern (*n* = 293, 47.7%)	*P* value
Age (years)	65.2 ± 12.6	64.6 ± 13.4	65.7 ± 11.9	0.671
Male, n (%)	416 (67.7)	226 (73.0)	190 (64.8)	0.033
Family history of CAD, n (%)	222 (36.1)	109 (33.8)	113 (38,5)	0.876
Arterial hypertension, n (%)	390 (634)	189 (58.7)	201 (68.6)	0.043
Dyslipidemia, n (%)	365 (59.3)	178 (55.2)	187 (63.8)	0.057
Diabetes mellitus, n (%)	180 (29.2)	78 (24.2)	102 (34.8)	0.024
Smokers, n (%)	285 (46.3)	138 (42.8)	147 (50.2)	0.059
PAD, n (%)	165 (26.8)	129 (40.1)	136 (46.4)	0.101
CKD stage > III, n (%)	134 (21.7)	58 (18.0)	76 (25.9)	0.042
Previous PCI, n (%)	159 (25.8)	83 (25.7)	76 (25.9)	0.912
Previous AMI, n (%)	28 (4.5)	13 (4.0)	15 (5.2)	0.523
Diagnosis				
Non-STEMI/unstable angina, n (%)	174 (28.3)	78 (24.2)	96 (32.7)	0.050
Stable CAD, n (%)	441 (71.7)	244 (75.8)	197 (67.2)	0.050
CAD extent				
Single vessel, n (%)	333 (54.1)	188 (58.4)	145 (49.4)	0.038
Multivessel, n (%)	282 (45.9)	134 (41.6)	148 (50.6)	0.038
Target-vessel localization				
LM, n (%)	9 (1.4)	5 (1.6)	4 (1.4)	0.013*
LAD, n (%)	288 (46.8)	127 (39.4)	161 (54.9)	0.013*
LCX, n (%)	194 (31.6)	123 (38.2)	83 (28.3)	0.013*
RCA, n (%)	112 (18.2)	67 (20.8)	45 (15.3)	0.013*
Lesion characteristics				
Bifurcations, n (%)	54 (8.8)	21 (6.6)	33 (11.2)	0.083
Calcification (moderate–severe), n (%)	87 (14.1)	30 (9.3)	57 (19.4)	0.003
Lesion length (mm)	24.99 ± 11.61	22.65 ± 9.24	26.32 ± 10.39	0.001
Lesions ≥ 25 mm, n (%)	199 (32.4)	78 (24.2)	121 (41.3)	0.001
Stent (n. per lesions)	1.20 ± 0.54	1.17 ± 0.51	1.24 ± 0.56	0.388
Pre-dilatation, n (%)	567 (92.3)	280 (86.9)	287 (97.9)	0.042
Post-dilatation, n (%)	479 (77.9)	263 (81.6)	216 (73.7)	0.145
Average stent length (mm)	24.09 ± 11.61	20.76 ± 5.83	34.21 ± 17.74	< 0.001
Average stent diameter (mm)	3.18 ± 0.41	3.19 ± 0.39	3.17 ± 0.42	0.613
Use of intravascular imaging (no. of lesions, %)	144 (23.4)	66 (20.5)	78 (26.6)	0.047

*AMI* acute myocardial infarction; *CAD* coronary artery disease; *CKD* chronic kidney disease; *PAD* peripheral artery disease; *PCI* percutaneous coronary intervention; *LAD* left anterior descending artery; *LCX* left circumflex artery; *LM* left main; *RCA* right coronary artery; *STEMI* ST-elevation myocardial infarction. *After Bonferroni correction for multiple comparison

### Lesion and procedural characteristic

The average QVPi value was 0.69 ± 0.12 and the physiological pattern was interpreted as predominantly diffuse in 293 vessels (47.7%). The average dQFR/ds value was 0.04 ± 0.04 and a major gradient was detected in 351 vessels (57.1%).

Diffuse CAD pattern was more likely to be observed in the left anterior descending artery (54.9 vs. 39.4%; *p* = 0.013). Diffuse CAD had on average longer lesion length (26.32 ± 10.39 vs. 22.65 ± 9.24 mm; *p* = 0.001) and were more frequently either moderately or severely calcified, according to the angiographic evaluation (19.4 vs. 9.4%; p = 0.003) compared with focal CAD. Consistently, pre-dilatation was performed more frequently in the group with diffuse CAD (97.9 vs. 86.9%; p = 0.042). Intravascular imaging was used more frequently in case of diffuse disease compared to focal CAD (26.6 vs. 20.5%; *p* = 0.047). Vessels with diffuse pattern showed a higher MLD (1.34 ± 0.41 vs. 1.19 ± 0.33 mm; p = 0.001) and lower DS (49.8 ± 11.2 vs. 55.2 ± 11.0%; *p* = 0.001). Vessels presenting a focal pattern showed a higher rate of major gradients (70.4 vs. 42.7%; *p* = 0.001).

Overall, the length of implanted stents/scaffold was significantly higher in patients with predominantly diffuse disease (34.21 ± 17.74 vs. 20.76 ± 5.83 mm; *p* < 0.001).

Complete procedural characteristics are displayed in Table 1**.**

### Quantitative flow ratio assessment

Pre-PCI mean angiography-derived FFR was 0.73 ± 0.12 with 430 vessels (69.9%) deemed as flow-limiting. Post-PCI mean angiography-derived FFR was 0.94 ± 0.04, with 199 vessels (32.4%) deemed as having suboptimal post-PCI physiology.

**Fig. 1:**
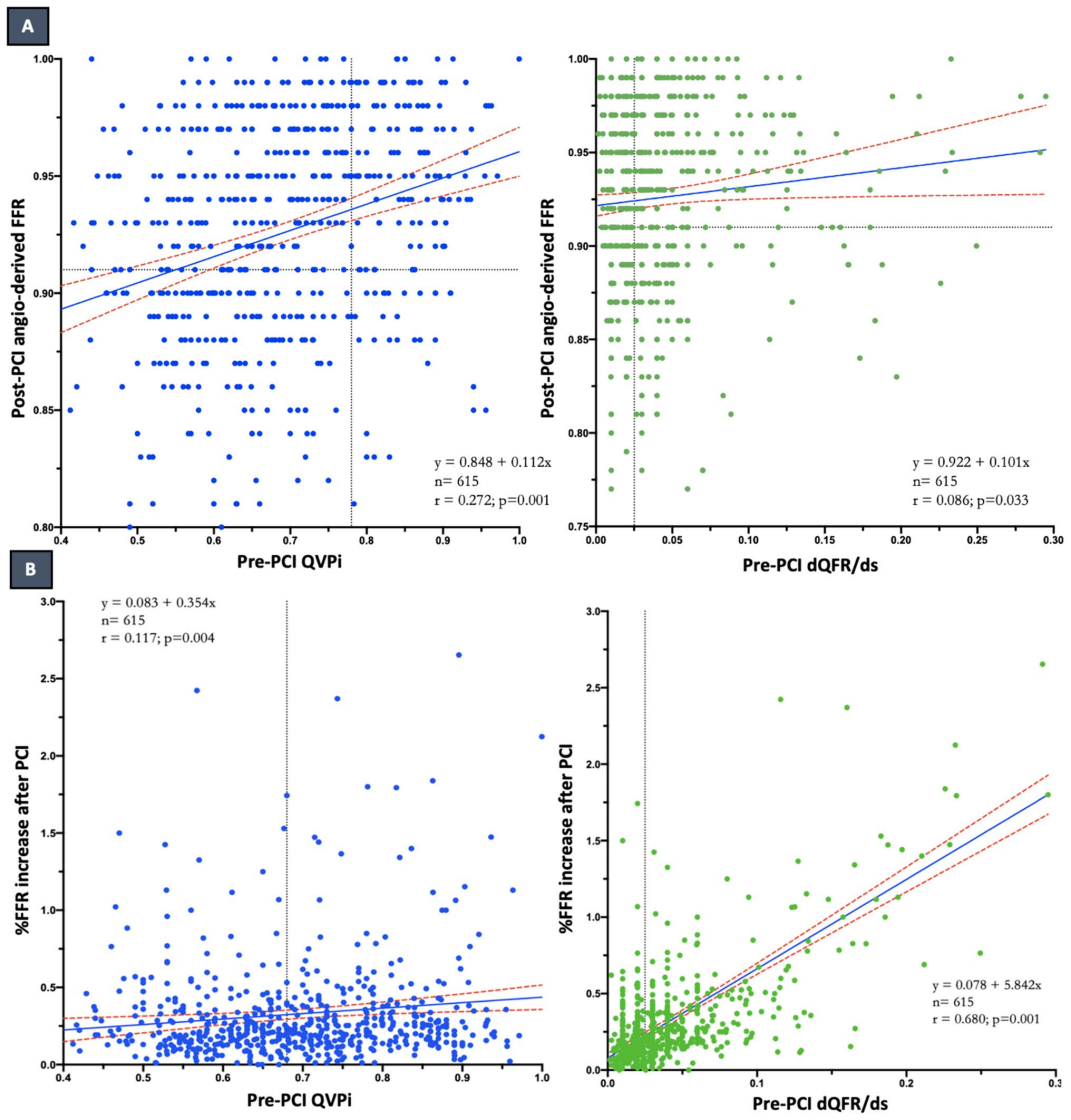
**A** Correlation between pre-PCI QVPi and post-PCI angiography-derived FFR (left) and between pre-PCI dQFR/ds and post-PCI angiography-derived FFR (right). **B** Correlation between pre-PCI QVPi and % μFR/QFR increase after PCI (left) and between pre-PCI dQFR/ds and % μFR/QFR increase after PCI (right). *PCI* percutaneous coronary intervention; *μFR* Murray law quantitative flow ratio; *QFR* quantitative flow ratio; *dQFR/ds* instantaneous QFR gradient per unit length; *QVPi* virtual pullback pressure gradient index

Pre-PCI QVPi showed a modest but significant correlation with post-PCI angiography-derived FFR (R = 0.272; *p* < 0.001), and weak correlation with the %FFR increase (R = 0.117; p = 0.004), while pre-PCI dQFR/ds showed no correlation with post-PCI angiography-derived FFR (R = 0.086; *p* = 0.033), and strong correlation with the %FFR increase (R = 0.680; p = 0.001), as displayed in Fig. 1.

**Fig. 2:**
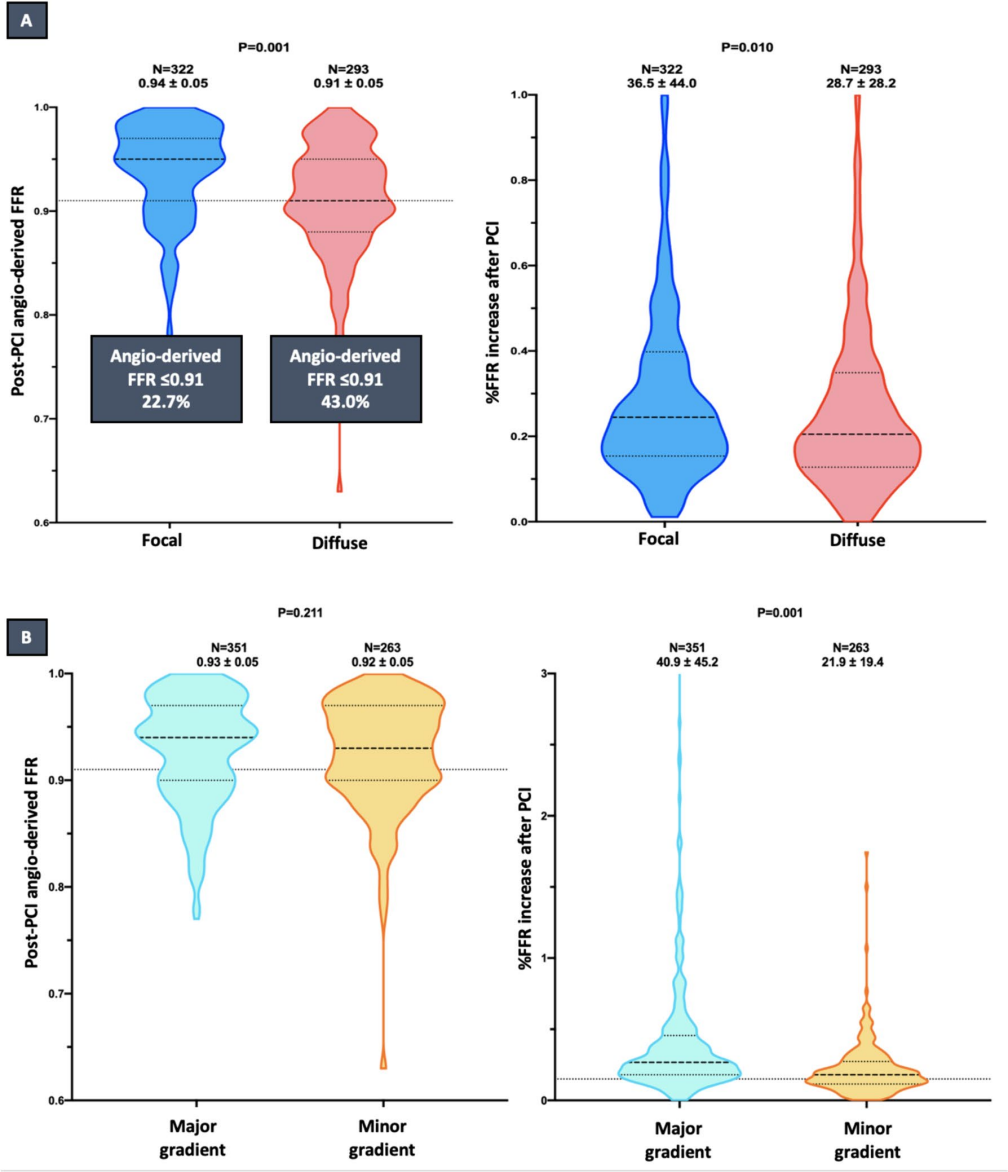
**A** Post-PCI μFR/QFR (left) and % μFR/QFR increase after PCI (right) according to physiological pattern of disease. **B** Post-PCI μFR/QFR (left) and % μFR/QFR increase after PCI (right) according to the presence of major drops at the dQFR/ds. *PCI* percutaneous coronary intervention; *μFR* Murray law quantitative flow ratio; *QFR* quantitative flow ratio; *dQFR/ds* instantaneous QFR gradient per unit length; *QVPi* virtual pullback pressure gradient index

Importantly, at completion of PCI, diffuse CAD showed worse post-PCI angiography-derived FFR values (0.91 ± 0.05 vs. 0.94 ± 0.05; *p* = 0.001) and percentage increase (28.7 ± 28.2 vs. 36.5 ± 44.0; p = 0.010) compared to focal with a higher rate of suboptimal physiological result (43.0 vs. 22.7%, *p* = 0.001), as shown in Fig. 2. Pre-PCI QVPi was inversely associated with the incidence of post-PCI angiography-derived FFR ≤ 0.91 (for 0.10 QVPi increase: OR 0.666; 95% CI 0.576–0.771; p = 0.001; supplementary Fig. 2). Angiography-derived FFR analysis data are reported in Table 2**.**

**Table 2:** Pre- and post-procedural angiographic and angio-derived characteristics

	Overall 615 vessels	Focal pattern 322 vessels	Diffuse pattern 293 vessels	*P* value
Pre-PCI				
Vessel angiography-derived FFR	0.73 ± 0.12	0.73 ± 0.13	0.72 ± 0.11	0.775
Vessel angiography-derived FFR ≤ 0.80, n (%)	430 (69.9)	276 (85.7)	154 (52.5)	0.001
RVD (mm)	2.54 ± 0.53	2.55 ± 0.49	2.55 ± 0.57	0.957
MLD (mm)	1.25 ± 0.37	1.19 ± 0.33	1.34 ± 0.41	0.001
DS (%)	53.09 ± 11.42	55.24 ± 11.01	49.81 ± 11.16	0.001
QVPi	0.69 ± 0.12	0.79 ± 0.08	0.58 ± 0.07	0.001
dQFR/ds	0.04 ± 0.04	0.06 ± 0.05	0.03 ± 0.02	0.001
Major drops, n (%)	351 (57.1)	226 (70.4)	125 (42.7)	0.001
Post-PCI				
Distal RVD (mm)	2.74 ± 0.51	2.75 ± 0.51	2.74 ± 0.53	0.881
MLD (mm)	2.51 ± 0.71	2.51 ± 0.56	2.53 ± 0.88	0.647
DS (%)	13.41 ± 11.91	13.81 ± 11.56	12.91 ± 12.39	0.441
QVPi	0.74 ± 0.11	0.77 ± 0.11	0.72 ± 0.11	0.001
dQFR/ds	0.01 ± 0.01	0.01 ± 0.02	0.01 ± 0.01	0.073
Vessel angiography-derived FFR	0.94 ± 0.04	0.94 ± 0.05	0.91 ± 0.05	0.001
Vessel angiography-derived FFR ≤ 0.91, n (%)	199 (32.4)	73 (22.7)	126 (43.0)	0.001
Acute functional gain	0.20 ± 0.13	0.21 ± 0.13	0.18 ± 0.12	0.002
Angiography-derived FFR % of increase post-PCI	32.8 ± 37.6	36.5 ± 44.0	28.7 ± 28.2	0.010
Angiography-derived FFR % of increase post-PCI < 15%, n (%)	165 (26.8)	76 (23.6)	89 (30.4)	0.055

*DS* diameter stenosis; *dQFR/ds* instantaneous QFR gradient per unit length; *MLD* minimal lumen diameter; *PCI* percutaneous coronary intervention; *QFR* quantitative flow ratio; *QVPi* QFR-virtual pullback index; *RVD* reference vessel diameter

### Intravascular imaging-guided PCI

Intravascular imaging was performed in 144 vessels (23.4%): 66 were classified as focal and 78 as diffuse (*p* = 0.047). IVUS was used in 49 (34.0%) vessels (20 focal vs. 29 diffuse; 40.8% vs. 49.2%, respectively; *p* = 0.127) and OCT in the remaining 97 (66.0%) vessels (41 focal vs. 56 diffuse; 42.3% vs. 57.7% respectively; *p* = 0.031). Clinical and procedural characteristics of vessel treated with intravascular imaging use are displayed in Supplementary Table 1.

No significant differences were noted in terms of post-PCI angiography-derived FFR values according to use of intravascular imaging both in diffuse and focal lesions. Pre-PCI QVPi showed a significant correlation with post-PCI angiography-derived FFR, regardless of the use of intravascular imaging (imaging: R = 0.196; p = 0.001; non-imaging: R = 0.341; *p* < 0.001), as displayed in Supplementary Fig. 3.

### Long-term outcomes

**Fig. 3:**
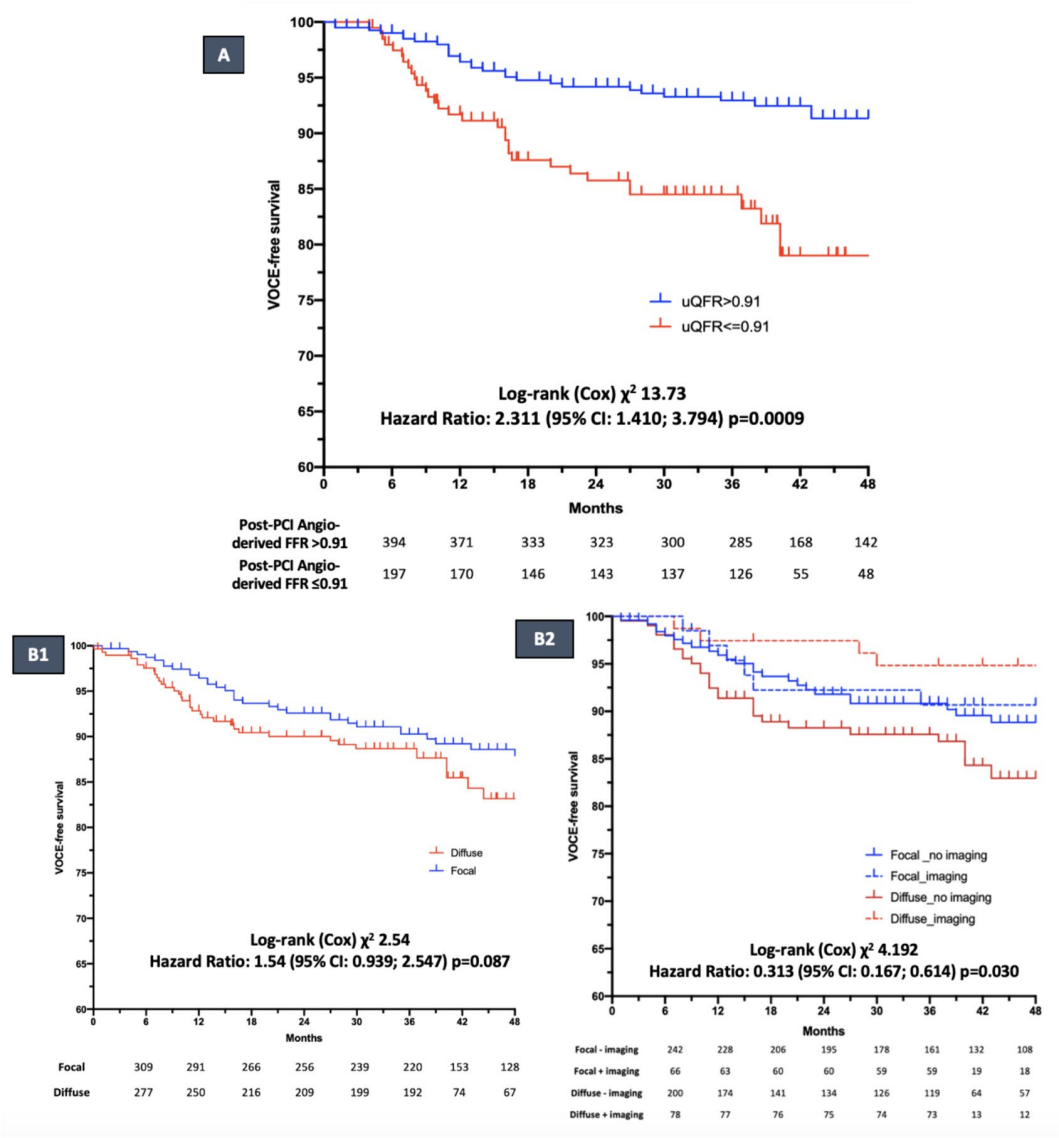
**A** Kaplan–Meier survival curves for 4 years VOCE according to sub-optimal post-PCI functional results (μFR/QFR ≤ 0.91). **B1** Kaplan–Meier survival curves for 4 years VOCE according to the physiological pattern of CAD. **B2** Kaplan–Meier survival curves for 4 years VOCE according to the physiological pattern of CAD stratified by the use of intravascular imaging. *PCI* percutaneous coronary intervention; *μFR* Murray law quantitative flow ratio; *QFR* quantitative flow ratio; *VOCE* vessel-oriented composite endpoint

At the median follow-up time of 37 months (35–60) VOCE occurred in 77 (12.7%) vessels, mainly driven by TVR (*n* = 66, 10.9%). Notably, patients with post-PCI suboptimal physiological result demonstrated a significantly higher risk of VOCE compared with patients with optimal physiological result in the shared frailty Cox regression model (16.2 vs. 7.6%; HR:2.311; 95% CI: 1.410–3.794; *p* = 0.0009), as displayed in Fig. 3A. Conversely, no significant difference in terms of VOCE incidence was reported according to the disease pattern (focal 8.8% vs. diffuse 12.2% HR: 1.54; 95% CI 0.939–2.547; *p* = 0.087; Fig. 3B1. Patients with predominantly diffuse disease who underwent intravascular imaging-guided PCI experienced a significantly lower incidence of VOCE at long term compared with patients without intravascular imaging (5.1% vs 15.8%; HR: 0.313, 95% CI 0.167–0.614, p = 0.030; Fig. 3B2. Long-term outcomes according to disease pattern and immediate post-PCI functional results are displayed in Table 3. Kaplan–Meier curves are displayed in Fig. 3.

**Table 3:** Long-term outcomes according to disease pattern and use of intravascular imaging

	Overall (*n* = 606)	Focal pattern (*n* = 318, 52.5%)	Diffuse pattern (*n* = 288, 47.5%)	P value	Optimal, angiography-derived FFR > 0.91 (*n* = 409, 67.5%)	Suboptimal, angiography-derived FFR ≤ 0.91 (*n* = 197, 32.5%)	*P* value
Target-vessel MI, n (%)	13 (2.1)	8 (2.5)	5 (1.7)	0.583	10 (2.4)	3 (1.5)	0.562
TVR, n (%)	53 (8.7)	25 (7.9)	28 (9.7)	0.472	24 (5.9)	29 (14.7)	< 0.001
TLR, n (%)	21 (3.5)	8 (2.5)	13 (4.5)	0.190	9 (2.2)	12 (6.1)	0.018
Death, n (%)	22 (3.6)	8 (2.5)	14 (4.9)	0.134	11 (2.7)	11 (5.6)	0.102
Cardiac Death, n (%)	10 (1.7)	3 (0.9)	7 (2.4)	0.205	6 (1.5)	4 (2.0)	0.735
VOCE, n (%)	63 (10.4)	28 (8.8)	35 (12.2)	0.185	31 (7.6)	32 (16.2)	0.002

	Diffuse pattern (*n* = 288, 47.5%)			Focal pattern (*n* = 318, 52.5%)		
	No imaging (= 210)	Imaging (*n* = 78)	*P* value	No imaging (*n* = 252)	Imaging (*n* = 66)	*P* value
Target-vessel MI, n (%)	4 (1.9)	1 (1.3)	1.000	6 (2.4)	2 (3.0)	0.731
TVR, n (%)	24 (11.4)	4 (5.1)	0.122	20 (7.9)	5 (7.6)	1.000
TLR, n (%)	12 (5.7)	1 (1.3)	0.197	7 (2.8)	1 (1.5)	1.000
Death, n (%)	13 (6.2)	1 (1.3)	0.122	6 (2.4)	2 (3.0)	0.673
Cardiac Death, n (%)	7 (3.3)	0 (0.0)	0.195	3 (1.2)	0 (0.0)	1.000
VOCE, n (%)	31 (14.8)	4 (5.1)	0.026	23 (9.1)	5 (7.6)	0.811

*MI* myocardial infarction; *QFR* quantitative flow ratio; *TLR* target lesion revascularization; *TVR* target vessel revascularization; *VOCE* vessel oriented composite endpoints

Presence of major gradients (dQFR/ds ≥ 0.025/mm) was shown to have no impact on VOCE incidence at long term (7.9% vs. 12.1%; HR: 1.501; 95% CI: 0.879–2.562; *p* = 0.157) regardless from the use of intravascular imaging.

The type of prosthesis implanted (either BRS or DES) had no significant impact on both immediate post-PCI suboptimal physiology (10.8% vs. 10.2%; *p* = 0.885) and long-term VOCE occurrence (30.1% vs. 33.6% *p* = 0.452). Kaplan–Meier analysis for implanted stent and disease pattern are shown in Supplementary Fig. 4.

## Discussion

In this large, multicenter, international cohort of patients with CAD undergoing PCI, we observed that the physiological pattern of disease, defined by functional angiography-derived quantitative measures, was associated with the functional outcome of the intervention. In particular, patients with predominantly diffuse disease showed a significantly worse post-PCI angiography-derived FFR and percentage of FFR increase compared with patients with predominantly focal disease.

Moreover, suboptimal post-PCI physiology was associated with worse vessel-oriented clinical outcomes and diffuse disease showed a numerically but not significantly higher rate of adverse events. Notably, the use of intracoronary imaging mitigated the risk of VOCE in patients with diffuse coronary disease, yielding a significantly improved clinical outcome compared to interventions guided by angiography alone.

Longitudinal physiological vessel analysis is a powerful tool to enhance precision medicine in terms of accurate diagnosis, procedural planning, and tailored treatment. Diffuse longitudinal distribution of atherosclerosis may impair the effectiveness of PCI. Indeed, stenting is a segmental treatment which may produce suboptimal functional outcomes in case of long lesions.

Discrimination between focal and diffuse disease is based mostly on coronary angiography, which is, however, characterized by low accuracy [25]. μFR/QFR-virtual pullback curve can be derived from standard angiographic images without further instrumentation of the coronary artery allowing identification of the lesions responsible for pressure loss along the vessel and quantifying the functional gain achievable with PCI [27, 28]. Results from AQVA and AQVA-2 trials already confirmed superiority of QFR-based virtual PCI over angiography-based PCI with regard to post-PCI optimal physiological results [29, 30].

Shin et al. suggested angiography-derived physiological pattern of disease to be a major determinant of optimal PCI result. Moreover, diffuse coronary atherosclerosis showed a correlation with higher rate of target vessel failure at follow-up despite optimal functional post-PCI results [31]. A third study from Dai et al. confirmed the prognostic value of pre-PCI QFR-PPGi regardless the post-PCI results [32]. Of note, in a sub-analysis of the TARGET-FFR (Trial of Angiography vs. pressure-Ratio-Guided Enhancement Techniques-Fractional Flow Reserve) study, diffuse CAD defined based on PW-pullback PPGi was seen to be associated with a higher rate of residual angina after successful PCI, as compared to focal disease (51.9% vs. 27.5%; *p* = 0.020) [5]. Our findings confirmed the impact of baseline QVPi on immediate functional PCI results. However, pre-PCI QVPi was not significantly associated with the risk of VOCE. Beside the longer follow-up, a possible reason for this divergence compared with the previously available evidence may be related to use of intravascular imaging that was performed in roughly a quarter of cases in our cohort.

Indeed, upfront characterization of the vessel may predict the risk of unsuccessful intervention and the need for additional tools such as intracoronary imaging.

The potential advantage of intracoronary imaging-guided PCI in case long coronary lesions has been already supported in several studies. A meta-analysis involving seven randomized trials (3,192 participants) demonstrated the superiority of IVUS-guided PCI compared to angiography alone in cases of long lesions, leading to a reduction in the risk of intra-stent restenosis and TLR by 79% [21]. Similarly, findings from the IVUS-XPL (Impact of Intravascular Ultrasound Guidance on the Outcomes of Xience Prime Stents in Long Lesions) and ULTIMATE (Intravascular Ultrasound-Guided Drug-Eluting Stents Implantation in “All-Comers” Coronary Lesions) trial revealed a decreased risk of major adverse cardiovascular events (5.6 vs. 10.7%; HR: 0.50; 95% CI 0.34–0.75; *p* < 0.001) and target lesion failure (6.6 vs. 10.7%; *p* = 0.01), respectively, in patients with long coronary lesions who underwent IVUS—compared to angiography-guided PCI [22, 23]. Also, in the RENOVATE-COMPLEX PCI (Randomized Controlled Trial of Intravascular Imaging Guidance versus Angiography-Guidance on Clinical Outcomes after Complex Percutaneous Coronary Intervention) trial, angiography-defined diffuse disease (lesion length > 38 mm) acknowledged for the 54% of the cases included [24]. In this trial, the use of intracoronary imaging led to a reduction of MACE (7.7 vs. 12.3%; HR: 0.64; 95% CI 0.45 to 0.89; *p* = 0.008), as compared to angiography alone. To the best of our knowledge, our study is the first to investigate the role of intravascular imaging according to the specific endotype of physiological pattern of disease. Notably, the use of either IVUS or OCT to optimize PCI seems to be associated with a better functional result and improved clinical outcomes, especially in patients with predominantly diffuse disease.

## Limitations

Our findings must be interpreted considering some limitations. First, this was a retrospective analysis of a multicenter international pooled cohort of patients. Moreover, the event adjudication was not performed by a dedicated independent event adjudication committee. Therefore, even though all the data collection was performed prospectively, reporting bias could not be excluded.

Second, variability in baseline clinical and procedural characteristics of the study sub-populations must be acknowledged. In particular, PCI was performed with BRS in a subgroup of patients enrolled in Verona. Furthermore, patients enrolled in the FORZA trial were randomized to OCT-guided vs FFR-guided PCI.

Third, cut-off values of the QVP index to define CAD patterns are not established and, in this study, we used the QVP median value to discriminate between predominantly focal vs predominantly diffuse pattern of disease. Moreover, it must be acknowledged that QVP index and dQFR/ds provide quantitative metrics to describe the pattern of disease and the local severity of CAD, but these indices do not inform on the presence of serial lesions or mixed patterns of disease.

Fourth, suboptimal angiographic quality may have impacted on the accuracy of angiography-derived coronary physiology measures. However, all the coronary imaging has been reviewed by expert QFR-certified operators blinded to the clinical data who have excluded low-quality angiograms.

In view of these limitations, our findings should be considered hypothesis-generating and they should be confirmed in a larger cohort of patients undergoing prospective assessment of the disease pattern.

## Conclusions

Functional angiography-derived longitudinal vessel analysis is a major determinant of post-PCI functional result. Suboptimal post-PCI coronary physiology is observed more often in coronary arteries with diffuse disease (low QVP) and no major gradient (low dQFR/ds), and it is associated with higher risk of vessel-oriented adverse events at long-term follow-up. In patients with diffuse disease intracoronary imaging may lower the risk of adverse events reducing the risk of unscheduled target vessel revascularization.

## Supplementary Information

Below is the link to the electronic supplementary material.

Supplementary file1 (DOCX 687 KB)

## Data Availability

The data that support the findings of this study are available from the corresponding author upon reasonable request.
