# Supplementary material for: Angiography-derived physiological patterns of coronary artery disease: implications with post-stenting physiology and long-term clinical outcomes
Source: Clin Res Cardiol. 2024 Aug 5;113(12):1745–56. doi: 10.1007/s00392-024-02500-8 (PMC11579185; doi:10.1007/s00392-024-02500-8)
Supplement: Supplementary file 1 — Supplementary file1 (DOCX 687 KB) [file 392_2024_2500_MOESM1_ESM.docx]

**Murray-law’s based QFR computation**

After selecting the angiographic view with optimal lesion exposure, minimal vessel overlap and foreshortening, the lumen contour of the interrogated coronary artery was automatically delineated, whilst the contrast flow velocity was derived from the length of the vessel centerline divided by the contrast filling time, and then converted into hyperaemic flow velocity. Time variable needed for flow velocity computation was derived by the angiographic acquisition’s frame rate and automatic frame counting. Subsequently, a frame with good contrast filling and full exposure of the lumen contour was selected as the analysis frame and the lumen boundaries of both the interrogated vessel and major side branches were delineated automatically. Lumen contour was automatically detected and the reference vessel diameter reconstructed considering the step-down phenomenon across bifurcations based on Murray bifurcation fractal law. Finally, pressure drop was calculated based on fluid dynamic equations with the above-mentioned hyperaemic flow as the boundary condition.

**Quantitative flow ratio computation**

QFR was computed using end-diastolic frames of 2 optimal angiographic projections that were separated by at least 25, a 3-dimensional model of the segment of interest and its reference vessel was constructed in an automated manner. Correction of vessel contours was manually performed by investigators if necessary. Distal segment of the target vessel was selected for the corresponding point of vessel QFR. In order to obtain contrast QFR value, estimated contrast coronary flow was calculated using TIMI frame-count adjustment along vessel extension as demarcated by its ostial part up to selected distal part.

| **CLINICAL FEATURES** | **Intravascular Imaging**  **(n=144, 23.4%)** | Non-Intravascular Imaging  (n=471, 76.6%) | P value |
| --- | --- | --- | --- |
| Age (years) | 68.2 ± 11.5 | 64.6 ± 12.5 | 0.104 |
| Male, n (%) | 88 (61.1) | 328 (69.6) | 0.098 |
| Previous PCI, n (%) | 48 (33.3) | 111 (23.6) | 0.034 |
| Stable CAD, n (%) | 101 (70.1) | 340 (72.2) | 0.834 |
| Multivessel, n (%) | 85 (59.0) | 197 (41.0) | 0.012 |
| Lesion Length (mm) | 23.88 ± 10.45 | 24.56 ± 10.56 | 0.675 |
| Stent (n. per lesions) | 1.19 ± 0.87 | 1.21 ± 0.76 | 0.546 |
| Pre-dilatation, n (%) | 131 (90.1) | 440 (93.4) | 0.782 |
| Post-dilatation, n (%) | 124 (89.5) | 350 (74.3) | 0.039 |
| Average stent length (mm) | 23.34 ±9.72 | 26.72 ± 11.34 | 0.067 |
| Average stent diameter (mm) | 3.18 ± 0.56 | 3.18 ± 0.79 | 0.987 |
| **PRE PCI** |  |  |  |
| Vessel QFR | 0.75 ± 0.24 | 0.72 ± 0.38 | 0.197 |
| RVD (mm) | 2.62 ± 0.48 | 2.52 ± 0.23 | 0.104 |
| MLD (mm) | 1.39 ± 0.41 | 1.17 ± 0.23 | 0.003 |
| QVPi | 0.64 ± 0.16 | 0.71 ± 0.13 | 0.011 |
| dQFR/ds | 0.04 ± 0.03 | 0.06 ± 0.04 | 0.389 |
| **POST PCI** |  |  |  |
| RVD (mm) | 2.69 ± 0.37 | 2.74 ± 0.49 | 0.591 |
| MLD (mm) | 2.59 ± 0.56 | 2.56 ± 0.38 | 0.311 |
| QVPi | 0.77 ± 0.13 | 0.79 ± 0.09 | 0.781 |
| dQFR/ds | 0.01 ± 0.01 | 0.01 ± 0.01 | 1.000 |
| Vessel QFR | 0.93 ± 0.05 | 0.95 ± 0.05 | 0.789 |

**Supplementary Table 1. Baseline clinical and procedural characteristics according to intravascular imaging use**

**Supplementary Figure 1. Flow-chart used for patients’ enrollment.**


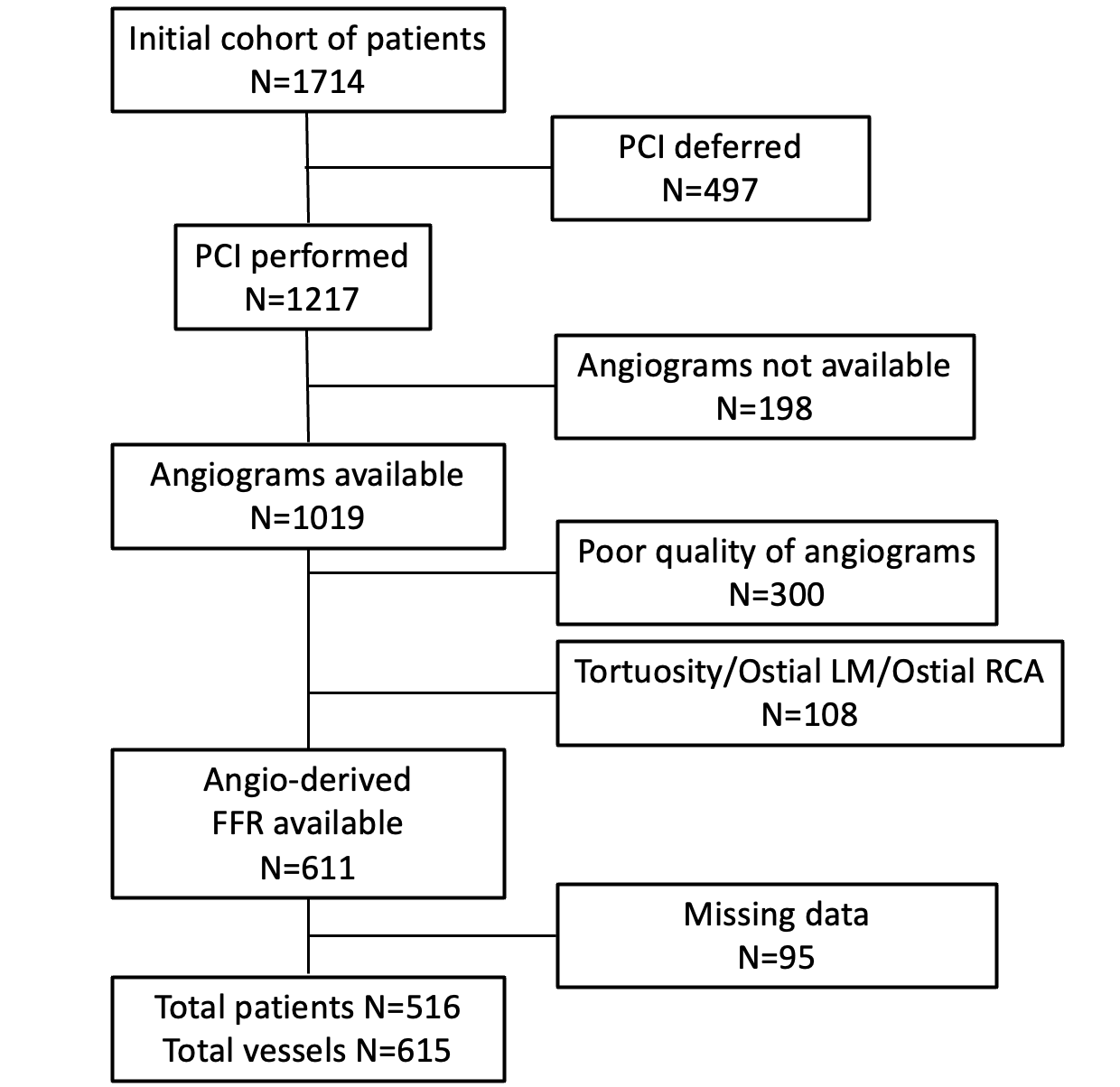


**Supplementary Figure 2. Spline Line for probability of suboptimal PCI results across whole range of prePCI QVPi values**


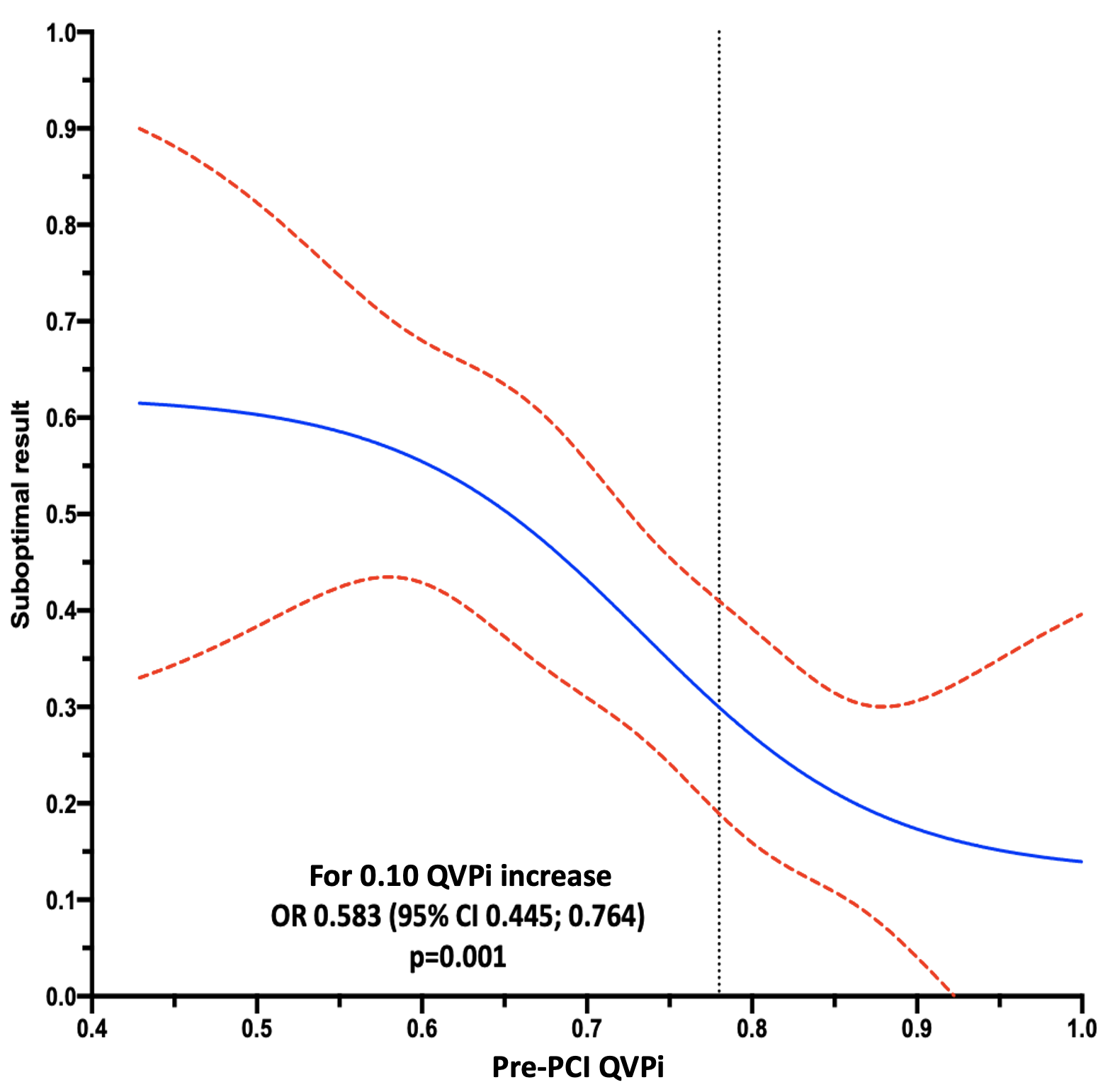


**Supplementary Figure 3. Scatter Matrix for Correlation of QVPi Pre and Post-PCI QFR values according to disease pattern and use of intravascular imaging.**


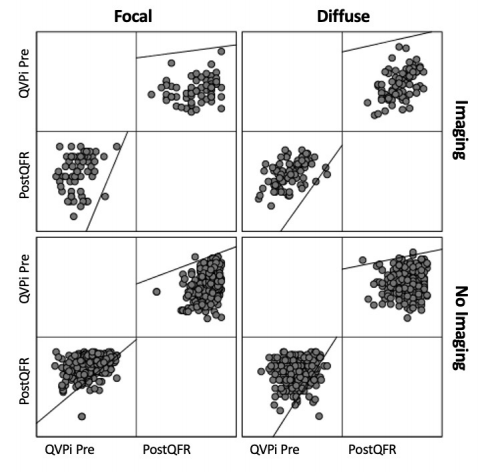


**Supplementary Figure 4. Immediate and long-term outcomes according to type of stent implanted and disease pattern.
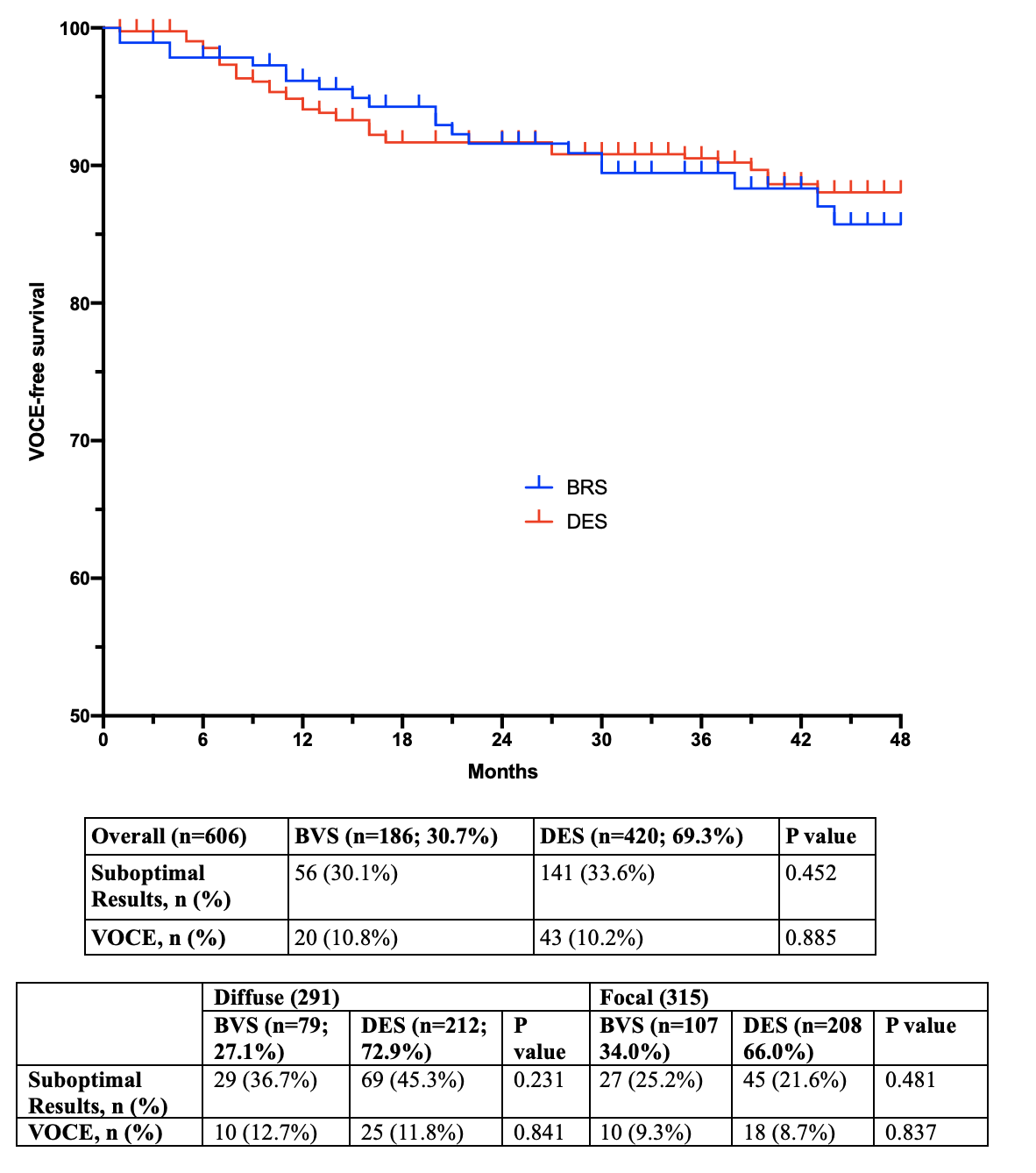
**
